# Transcranial magnetic stimulation and intravenous ketamine combination therapy for treatment-resistant bipolar depression: A case report

**DOI:** 10.3389/fpsyt.2022.986378

**Published:** 2022-09-21

**Authors:** Laurent Elkrief, Olivier Payette, Jean-Nicolas Foucault, Christophe Longpré-Poirier, Maxime Richard, Véronique Desbeaumes Jodoin, Paul Lespérance, Jean-Philippe Miron

**Affiliations:** ^1^Centre Hospitalier de l’Université de Montréal et Centre de Recherche du CHUM, Université de Montréal, Montreal, QC, Canada; ^2^Département de Psychiatrie et d’Addictologie, Faculté de Médecine, Université de Montréal, Montreal, QC, Canada

**Keywords:** TMS, rTMS, TRD, IV ketamine, case report

## Abstract

About a third of patients suffering from major depression develop treatment-resistant depression (TRD). Although repetitive transcranial magnetic stimulation (rTMS) and intravenous ketamine have proven effective for the management of TRD, many patients remain refractory to treatment. We present the case of a patient suffering from bipolar TRD. The patient was referred to us after failure to respond to first-and second-line pharmacotherapy and psychotherapy. After minimal response to both rTMS and ketamine alone, we attempted a combination rTMS and ketamine protocol, which led to complete and sustained remission. Various comparable and complimentary mechanisms of antidepressant action of ketamine and rTMS are discussed, which support further study of this combination therapy. Future research should focus on the feasibility, tolerability, and efficacy of this novel approach.

## Introduction

Despite the available first-line therapies for major depression, around 1/3 of patients will develop treatment-resistant depression (TRD) (1, 2). In those cases, repetitive transcranial magnetic stimulation (rTMS) (3) and IV racemic ketamine (4) have shown clear effectiveness. Still, many patients will remain refractory even to these advanced approaches, and electroconvulsive therapy will usually be the next step. Given the issues related to patient acceptability of ECT (5), other options sometime need to be considered. As such, it has been proposed that a combination of TMS and ketamine could prove effective in TRD. Indeed, Best and colleagues reported long-lasting improvement in TRD patients treated with a combination of TMS and IV ketamine, even in those for which these individual approaches had limited effectiveness (6). This interesting study demonstrated the effectiveness of a unique rTMS and ketamine combination strategy where patients received relatively high doses of ketamine (0.4–2.3 mg/kg) on average, over 20 min, while simultaneously receiving low frequency rTMS for 30 min. In the context of the limited availability of literature of combination strategies, we present the case of a patient suffering from bipolar TRD who initially had limited improvement to rTMS and IV ketamine individually, but who experienced complete and lasting remission following a novel combination of theta-burst stimulation rTMS therapy and standard dose (0.5 mg/kg) IV ketamine therapy. The patient gave his consent to the publication of his case report.

## Case report

The patient was a 43-year-old male who had been suffering from recurrent depression since early adulthood and who had been diagnosed with bipolar disorder type I in 2017, following his first and only manic episode. Since then, he was suffering from severe intractable TRD. The patient had been a high functioning individual, holding a Ph.D and an executive position for many years before his manic episode. He was now without a job and presenting minimal functioning, living in his parent’s basement. Treatment history included several years of psychotherapy and the unsuccessful trial of various antidepressants, antipsychotics, mood-stabilizers, and psychostimulants of adequate dosage and duration. The patient was also suffering from comorbid generalized anxiety disorder. Past medical history was significant for low testosterone treated with testosterone gel and restless leg syndrome previously treated with pramipexole.

**FIGURE 1:**
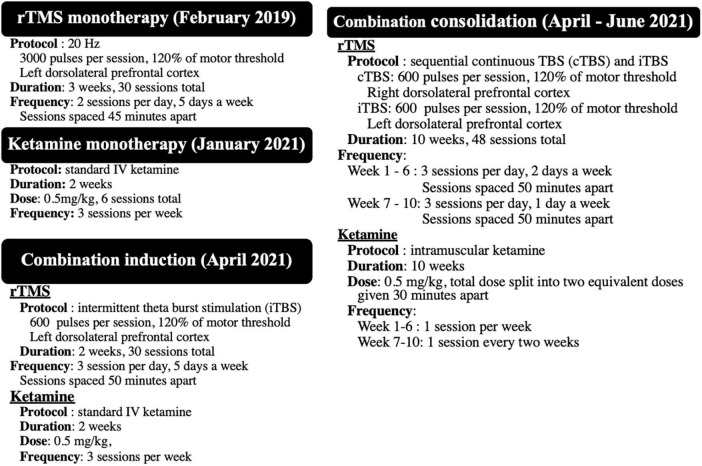
Summary of the four different treatment protocols received between 2019 and 2021.

The patient was initially referred for rTMS in January of 2019. At that time, baseline Montgomery-Asberg Depression Rating Scale (MADRS) was at 38 and Clinical Global Impression (CGI) at five. At baseline, the patient was receiving, quetiapine XR 300 mg HS, quetiapine 25 to 50 mg OD PRN, zolpidem 10 mg HS, clonazepam 1 mg AM and 2 mg HS, venlafaxine 262.5 mg OD and bupropion XL 150 mg OD. In February 2019, the patient underwent a first trial of rTMS in which he received two daily sessions (spaced by 45 min) of a 20 Hz, 3,000 pulses protocol (5 sec ON, 25 sec OFF) over the left dorsolateral prefrontal cortex (DLPFC) using the adjusted BeamF3 algorithm, delivered using a MagPro X100 device with a Cool-B70 coil (MagVenture, Farum, Denmark) at 120% of resting motor threshold (MT) (7). Treatment was delivered every weekday for a total of 30 sessions over 3 weeks. The patient unfortunately did not show signs of improvement, scales remaining similar to baseline value (Figure 1). Of note during this phase of treatment was that the patient remained on 3 mg of clonazepam per day throughout treatment, which he was unable to wean off because of crippling anxiety.

The patient was referred to us again in September 2020 for ketamine given his hesitancy to proceed with ECT. After a slow decrease of his clonazepam over several months down to 0.25–0.5 mg HS, the patient underwent IV ketamine therapy, in January of 2021. The patient received three infusions, each week, of IV ketamine at 0.5 mg/kg over 60 min for 2 weeks. MADRS went from 30, at treatment initiation, down to 17 on the last day of treatment but went back up to 23, 1 week after treatment, and he experienced full relapse with MADRS at 35, 4 months later.

Given his refusal to proceed with ECT, we offered an intensive combined rTMS/ketamine therapy according to the below described protocol over a 2-week period. At the time, the patient was receiving the following medication regimen: quetiapine 50 mg TID, clonazepam 0.5 mg HS, bupropion XL 150 mg OD, trazodone 50–15 0 mg HS PRN, and pramipexole 0.25 mg HS. On each weekday afternoon, the patient received three daily sessions (50 min between each session) of standard intermittent theta burst stimulation (iTBS) at 600 pulses per session at 120% MT over the left DLPFC (8), located using the adjusted BeamF3 algorithm (9). Additionally, three mornings per week over those same 2 weeks, he received the same IV ketamine protocol as before (six sessions total). IV ketamine treatments and rTMS treatments were spaced by 4 h (ketamine in the morning, rTMS in the afternoon). The patient initially experienced partial improvement on his last day of treatment, MADRS decreasing from 35 to 23, Hamilton Depression Scale 17 (HAMD-17) scores from 28 to 11, Patient Health Questionnaire-9 (PHQ-9) scores from 21 to 7, Generalized Anxiety Disorder-7 (GAD-7) scores from 17 to 9 and CGI from 5 down to 4. Given the positive response, we continued with a 10-week consolidation phase, during which the patient received 2 days of rTMS (three sessions per day, spaced 50 min apart)administered every week for 6 weeks, and then 1 day per week (three sessions per day, spaced 50 min apart) for four additional weeks (48 treatment sessions total). Each rTMS session began with a 2 min, 600 pulse, 100% MT, right DLPFC continuous TBS (cTBS) treatment (10), directly followed by the above describe iTBS protocol. cTBS was delivered over the right DLPFC with the coil centered on the F4 EEG location (using the BeamF3 algorithm, but on the right-side of the head) (11). During the consolidation phase of treatment, the patient received intramuscular (IM) racemic ketamine (same dosage as IV) once a week for 6weeks and then once every 2 weeks (eight sessions total). The IM ketamine was administered in the morning, and total dose (0.5 mg/kg) was split into two equivalent doses given 30 min apart. On days where patient received IM ketamine, ketamine was received in the mornings, and rTMS treatment was given in the afternoon (treatments spaced by 4 h). Throughout the consolidation phase, the patient continued to experience additional clinical improvement, eventually reaching full and sustained remission. During the consolidation phase, minor adjustments were done to his medication regimen: low dose pramipexole was added back to better control his restless leg syndrome, and clonazepam was increased back to 1.5 mg after the end of the consolidation phase to better control anxiety. At the end of the consolidation phase, the patient scored a four on MADRS, one on CGI a HAM- D-17 of five, PHQ-9 of one and GAD-7 of one. At the last follow-up, December 2021–close to 6 months after the last consolidation treatment–the patient had fully re-integrated his work functions, had moved out from his parent’s home, and had a MADRS of two, CGI of one, HAM-D-17 of three, PHQ-9 of three, and GAD-7 of 0.

**FIGURE 2:**
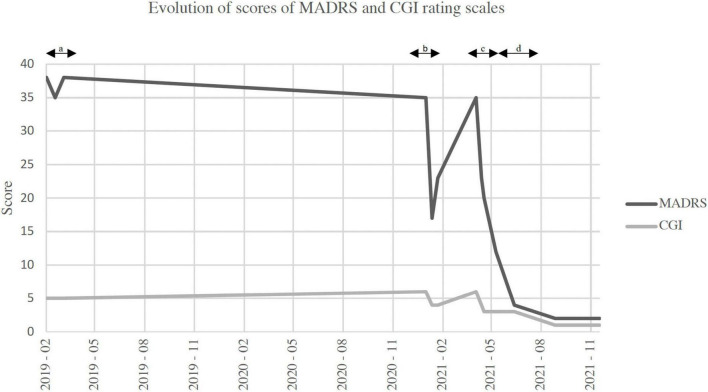
Evolution of scores of Montgomery-Asberg depression rating scale (MADRS) and clinical global impression (CGI) rating scales. **(A)** Duration of repetitive transcranial magnetic stimulation (rTMS) monotherapy. **(B)** Duration of ketamine monotherapy. **(C)** Duration of combination induction phase. **(D)** Duration of combination consolidation phase. MADRS, Montgomery-Asberg depression rating scale; CGI, clinical global impressions scale.

**FIGURE 3:**
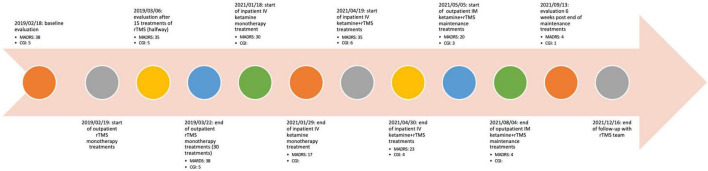
Detailed timeline of significant events and scores on rating scales.

See Figure 1 for a summary of the three phases of treat. See Figure 2 for timeline of events. See Figure 3 for a graphical representation of MADRS and CGI scores over follow-up.

## Discussion

We have presented the case of a patient suffering from bipolar TRD with limited improvement to rTMS and IV ketamine individually, but who experienced complete and sustained remission with combination therapy. Upon consultation in 2019, the patient was initially offered a high-frequency 20 Hz rTMS protocol, which has been demonstrated to be comparable to the FDA-approved 10 Hz and sham protocols (12). This initial rTMS trial did not show any clinical effects. Of note was that the patient was taking 3 mg of clonazepam per day, for which the patient was unable to be weaned off at the time. This is notable considering the evidence that benzodiazepines have been shown to reduce the effectiveness of rTMS (13) and ketamine treatments (14), in those suffering from depression. As such, the patient was asked to reduce his benzodiazepine dose before initiating his first trial of ketamine. Considering the lack of sustained response to ketamine monotherapy, the combination of ketamine and rTMS was suggested. During this phase of treatment, the rTMS protocol was changed to an iTBS protocol, considering the accumulating evidence of effectiveness of iTBS in treatment resistant patients (8), as well as the advantages of shorter treatment duration in regard to cost-effectiveness (15). In view of the patient’s strong response to combination treatments, as well as his history of rapid relapse, a maintenance phase was offered. In this phase, the cTBS protocol was added phase to better control anxiety symptoms (10). Seeing that there are currently no evidence-based protocols for rTMS or ketamine maintenance therapy, the patient was offered our clinics current protocol 10-week protocol.

Ketamine and rTMS are two evidence-based strategies in the management of treatment resistant depression (4, 16). Recently, two studies have examined the comparative effectiveness of these treatments in controlled (17) and naturalistic settings (18). Moreover, one group has published a series of a retrospective review of a form of combined rTMS and ketamine protocol (6). These initial results reported by Best and colleagues are promising, demonstrating a sustained statically significant mean reduction in CGI severity (4.46 ± 0.54, *p* < 0.0001), over a 2-year period (6). However, their protocol differs greatly than the one described here. In the Best et al. study (6), the patients received three daily sessions of low frequency rTMS, whereas we used TBS. Moreover, in their study, Best et al. used much higher doses of IV ketamine (close to four times higher doses). Finally, patients received ketamine and rTMS concomitantly, whereas our patient received these treatments sequentially. Nevertheless, taken together with the results reported by Best and colleagues, the sustained clinical response to our combination protocol, although greatly limited as a single case report, does merit further investigations into potential standardized combination approaches.

It should also be noted that, while ketamine and rTMS have similar clinical effectiveness and tolerability, some of the proposed antidepressant mechanisms of action of both treatment modalities are seemingly complimentary. At the molecular level, one of the proposed mechanisms of ketamine’s antidepressant effect is the “disinhibition hypothesis” (19), which posits that ketamine preferably binds to NMDAR on GABAergic interneurons, leading to disinhibition and overall increase in glutamatergic neurotransmission in relevant prefrontal cortical structures (19). Moreover, ketamine has been shown to promote neural plasticity, in cortical and subcortical structures, potentially through increase in neurotrophic factors such as brain-derived neurotrophic factor (BDNF) and mammalian target of rapamycin (mTOR) (20). These actions are vital, as current models of depression suggest that depression develops through chronic stress-mediated decreases in BDNF and subsequent reduction in plasticity in medial prefrontal cortex and hippocampus (21). The literature also suggests that iTBS could consolidate and stabilize acute ketamine induced neuroplasticity which would explain why the antidepressant effects lasted for more than what is typical for ketamine treatments (4). At the network level, it has been proposed that depression develops through abnormal default mode network (DMN) activity (22, 23), or aberrant functional connectivity between DMN and other networks such as salience network, central executive network, and various sensory networks (23, 24). Interestingly, there is recent evidence suggesting an effective reduction of the aberrant connectivity of the default mode network (DMN) and salience networks (SN) in depressed patients in studies using either iTBS protocols (25) or IV ketamine (26). Also, it has been shown that combining rTMS to psychotherapy helps improve both treatments efficacy in treating MDD by the activation of similar cortical circuits by both treatments (27). Here, ketamine might have had a similar role by inducing neural plasticity in prefrontal and limbic regions (21) which then allowed iTBS to effectively modulate DMN aberrant functional connectivity (28). Lastly, ketamine can also normalize activity in the orbitofrontal cortex and thus potentially impact depressive ruminations, an area not targeted by standard TMS (29, 30).

Limitations of this case report include the potential effects of reduction of clonazepam doses on the response to rTMS and ketamine combination therapy; however, clonazepam had already been reduced during ketamine monotherapy, which did not lead to clinically significant effects. rTMS protocol changes from 20 Hz to TBS could be responsible for the overall improvement, although both are thought to have similar effects (8), and switching between modalities was recently shown to only bring about modest additional improvement (31). The addition of pramipexole during the maintenance phase of the patient’s combination therapy may have contributed to sustained remission; however, the patient had received this medication in the past without any benefits, and the doses used we far below what is proposed for TRD (32).

In conclusion, the potentially synergic effect of ketamine with adequate rTMS treatment may have allowed for a complete and sustained remission of depressive symptoms in this seemingly hyper-refractive patient. These results, along with previous case-reports, suggest the potential role of TMS and IV ketamine combination therapy and the need for additional studies.

### Patient perspective

I have experienced at least four episodes of unipolar major depressive disorder since age 18 and they progressively lasted longer and longer with the most recent two episodes lasting more than 2 years. As described in the case report, the depressive episode I began experiencing at age 43 was by far the most severe and debilitating of all.

This was in part due to the fact that it was preceded by my first and only manic episode which was highly destructive to my social, professional, and personal life. I felt suicidal, ashamed, hopeless and helpless, and highly anxious to the point of isolating myself in my parents home and fearing going outside for over a year, refusing psychotherapy, believing it was now pointless. After nearly 20 years of independent life and working as a licensed health-care professional, researcher, and director of treatment programs in New York City, the compounded shame and grief of “having lost everything” in addition to the depression itself left me feeling unrecognizable to myself and others and afraid to seek support from my ever diminishing network of friends.

Both my personal psychiatrist and primary care doctor had been encouraging me to accept inpatient residential treatment and electroconvulsive therapy. As desperate as I was, I was adamant not to accept this treatment plan.

Instead, having been aware of rTMS and ketamine therapy for many years, I mustered up every last bit of willpower to advocate for myself and requested a referral from my psychiatrist to a clinic that might be able to provide one of these less invasive but promising forms of treatment.

Upon being accepted as a patient for my first round of rTMS treatment I began feeling some hope. But after several weeks of treatment and seeing little to no relief from symptoms, my hopes were dashed. Frustrated and defeated, I returned to spending all my time confined in my parents’ home.

I did, however, agree to begin psychodynamic psychotherapy, and a time-limited Acceptance and Commitment Therapy Group. I found the latter minimally effective and difficult to engage with due to my own clinical biases, the didactic approach of the therapists and my own reticence to identify with the other patients in the group.

About 9 months after the first rTMS treatments I asked for a second referral to the treatment team and expressed a desire for ketamine treatment.

The team at CHUM offered a first ketamine treatment, and my symptoms improved rapidly (within hours) following the first ketamine treatment. After the first full course of ketamine treatment I started going outside, exercising, driving, taking better care of my hygiene and reaching out to a small number of friends. While the effect was profound it began to wane after several weeks. As such, upon follow-up assessment I was offered the rTMS-ketamine combination treatment described in the above case report.

Although I was hoping to have a series of breakthrough “peak psychedelic experience” on ketamine which I believed would lead to a shift in perspective, profound catharsis, and deep emotional processing, this was not the subjective effect for me during medication infusions.

At times I felt intense dissociation and some nausea and other times hardly anything at all other than relaxation as I listened to my own meditative playlist during medication infusions. At other times, the immediate effect was unpleasant but it was usually followed by a sense of symptom relief. I should stress that at no point was there a desire to re-experience this drug effect recreationally.

During the rTMS treatments, I felt I was in good hands and trusted this protocol. The rTMS sessions did not feel invasive. I felt no side effects.

The combination treatment experience was similar to my previous rTMS and ketamine treatments, but upon discharge I finally felt enough motivation and symptom relief to begin making intense efforts to maintain the momentum on my own.

I went outside every single day and began planning my future, meeting old friends and making new ones. Relatively quickly I re-entered the workforce in private practice and even began dating again. The gains after that were progressive and long lasting.

As I write this today, over a year later, I feel full of gratitude and hope for my future. I am in a fulfilling relationship, traveling and working remotely in different cities, knocking off destinations on my bucket list. I feel grounded, ambitious and even playful at times. Although adherent to my medications, sometimes I fear a relapse, given my multiple experiences with treatment resistant depression.

However, knowing that there are innovative treatments out there, and in particular a combination approach that has worked well for me helps me feel more secure and optimistic that even though a relapse is possible that perhaps I would be able to access quick and efficient treatment in the future and for that I am thankful.

It is my hope that these treatments become more readily accessible to the public and more widely accepted by medical establishments.

## Data availability statement

The original contributions presented in this study are included in the article/supplementary material, further inquiries can be directed to the corresponding author.

## Ethics statement

Ethical review and approval was not required for the study on human participants in accordance with the local legislation and institutional requirements. The patients/participants provided their written informed consent to participate in this study.

## Author contributions

LE: conceptualization, writing–original draft, and review and editing. OP, MR, and J-NF: writing–original draft and review and editing. CL-P: data curation and writing–review and editing. VDJ: conceptualization, data curation, and writing–review and editing. PL: patient management and reviewing. J-PM: conceptualization, patient management, data curation, and writing–review and editing. All authors contributed to the article and approved the submitted version.
